# Expression of Selected miRNAs in Undifferentiated Carcinoma with Osteoclast-like Giant Cells (UCOGC) of the Pancreas: Comparison with Poorly Differentiated Pancreatic Ductal Adenocarcinoma

**DOI:** 10.3390/biomedicines12050962

**Published:** 2024-04-26

**Authors:** Alexey Popov, Jan Hrudka, Arpád Szabó, Martin Oliverius, Zdeněk Šubrt, Jana Vránová, Vanda Ciprová, Jana Moravcová, Václav Mandys

**Affiliations:** 1 Department of Pathology, 3rd Faculty of Medicine, Charles University, University Hospital Královské Vinohrady, 100 00 Prague, Czech Republic; alexey.popov@lf3.cuni.cz (A.P.); arpad.szabo@fnkv.cz (A.S.);; 2 Department of Surgery, 3rd Faculty of Medicine, Charles University, University Hospital Královské Vinohrady, 100 00 Prague, Czech Republic; martin.oliverius@fnkv.cz (M.O.); zdenek.subrt@fnkv.cz (Z.Š.); 3 Department of Medical Biophysics and Medical Informatics, 3rd Faculty of Medicine, Charles University, 100 00 Prague, Czech Republic; jana.vranova@lf3.cuni.cz; 4 Institute of Pathology, 1st Faculty of Medicine, Charles University, General University Hospital, 100 00 Prague, Czech Republic; 5 Clinical and Transplant Pathology Centre, Institute for Clinical and Experimental Medicine, 140 00 Prague, Czech Republic

**Keywords:** pancreas, undifferentiated carcinoma with osteoclast-like giant cells, ductal adenocarcinoma, miRNA

## Abstract

Undifferentiated carcinoma with osteoclast-like giant cells (UCOGC) of the pancreas represents a rare subtype of pancreatic ductal adenocarcinoma (PDAC). Despite a distinct morphology and specific clinical behavior, UCOGCs exhibit unexpected similarities in regard to DNA mutational profiles with conventional PDAC. Treating pancreatic ductal adenocarcinoma is particularly challenging, with limited prospects for cure. As with many other malignant neoplasms, the exploration of microRNAs (miRNAs, miRs) in regulating the biological characteristics of pancreatic cancer is undergoing extensive investigation to enhance tumor diagnostics and unveil the therapeutic possibilities. Herein, we evaluated the expression of miR-21, -96, -148a, -155, -196a, -210, and -217 in UCOGCs and poorly differentiated (grade 3, G3) PDACs. The expression of miR-21, miR-155, and miR-210 in both UCOGCs and G3 PDACs was significantly upregulated compared to the levels in normal tissue, while the levels of miR-148a and miR-217 were downregulated. We did not find any significant differences between cancerous and normal tissues for the expression of miR-96 and miR-196a in G3 PDACs, whereas miR-196a was slightly, but significantly, downregulated in UCOGCs. On the other hand, we have not observed significant differences in the expression of the majority of miRNAs between UCOGC and G3 PDAC, with the exception of miR-155. UCOGC samples demonstrated lower mean levels of miR-155 in comparison with those in G3 PDACs.

## 1. Introduction

In the latest WHO classification, undifferentiated carcinoma with osteoclast-like giant cells (UCOGC) of the pancreas is considered a rare subtype of pancreatic ductal adenocarcinoma (PDAC) [1], accounting for approximately 0.4% of pancreatic carcinomas [2]. The initial description of UCOGC was reported in 1954 [3]. Fourteen years later, Rosai labeled the lesion “carcinoma simulating giant cell tumor of bone” [4].

UCOGC exhibits a distinct morphology and typically carries a grim prognosis. The tumor comprises three main cell types: (1) neoplastic cytokeratin-positive mononuclear cells exhibiting cytonuclear atypia, (2) non-neoplastic mononuclear macrophages, and (3) non-neoplastic osteoclast-like multinucleated giant cells, which vary in quantity and are often accompanied by hemorrhage and necrosis. Despite their morphological differences, the DNA mutational profiles of UCOGCs surprisingly mirror those of conventional PDAC, frequently featuring *KRAS* activation and the inactivation of *SMAD4*, *TP53*, and *CDKN2A* [5,6,7,8]. Many neoplastic pathways in pancreatic cancer are regulated by microRNAs. However, little is known about miRNA expression profiles in UCOGCs and eventual differences in comparison with PDACs.

MicroRNAs (miRNAs) represent a group of single-stranded RNA molecules ranging from 15 to 27 nucleotides long. These compact, non-coding RNAs play pivotal roles in regulating various cellular processes within both healthy and cancerous cells. Functionally, miRNAs can bind to messenger RNAs (mRNAs), thereby modulating the translation process. Consequently, this interaction can lead to the downregulation of specific protein synthesis, serving as a crucial regulatory step in gene expression [9,10,11]. This post-transcriptional mechanism ensures precise control over essential cellular processes, including proliferation, migration, and apoptosis, which are fundamental to cancer growth, dissemination, and resistance to therapy. MiRNAs play a crucial role in the intricate interplay between cancer cells and other components within the tumor microenvironment. Given the challenging prognosis of PDAC, despite available treatments, there is considerable interest in studying the regulatory functions orchestrated by miRNAs in this malignancy. Such investigations aim to identify miRNAs as potential targets for innovative therapeutic strategies [12,13,14,15,16,17,18,19]. MiRNAs have been proposed to function as both oncogenes and tumor suppressors, and alterations in miRNA expression have been observed in neoplastic precursor lesions [20,21]. The dysregulation of miRNA expression profiles is a common feature in various cancer types, including PDAC [12,13,22]. Zhang et al. showed substantial variations in the relative expression levels of miRNAs across individual cases of pancreatic ductal adenocarcinoma, with changes spanning six orders of magnitude, ranging from 0.01 to 10,000 [23]. Aberrantly expressed microRNAs, documented across numerous studies, have been suggested as potential predictive factors for disease progression, chemotherapy response, and patient survival [24,25,26,27].

In this study, the expression of seven selected miRNAs (miR-21, miR-96 miR-148a, miR-155, miR-196a, miR-210, and miR-217), described to be deregulated in PDACs [28,29,30], was analyzed in UCOGC and poorly differentiated (G3) PDAC. In greater detail, these seven miRNAs were chosen due to their substantial involvement in pancreatic ductal adenocarcinoma, impacting tumor morphology, progression, and clinical prognosis. Notably, among the selected miRNAs, miR-21, miR-155, and miR-217 have been specifically associated with differential expression patterns correlated with tumor advancement [25,27,28]. MiR-21, miR-155, miR-196a, and miR-210 were chosen because they have been suggested as potential diagnostic and prognostic markers in previous studies [24,28,31]. Additionally, miR-96 and miR-148a were included in the selection due to their established roles as tumor suppressors in PDAC [32,33]. The aim of this work was to characterize the expression of these miRNAs in UCOGC and to identify possible differences in miRNAs expression between UCOGC and G3 PDAC.

## 2. Materials and Methods

### 2.1. Patients and Samples

A cohort of 10 patients (8 male, 2 female, aged 50–76 years) who underwent surgical treatment for pancreatic UCOGC and were histologically diagnosed in three participating institutions from 2003 to 2019 were included in the study. From those, nine were primary tumor resection specimens, and of those, four patients displayed regional lymph node metastasis at time of surgery. One case was a specimen of lymph node metastasis obtained from explorative laparotomy, without pancreas resection. There were two cases of UCOGC associated with conventional PDAC and one case associated with intraductal papillary mucinous neoplasm (IPMN); the remaining seven patients exhibited pure UCOGCs. The tumor size at surgery varied from 5mm to 140mm; two cases were pT1, two cases were pT2, five cases were pT3, and one case was pTX stage, respectively. Due to the rarity of UCOGC, we utilized cases from our previously published studies [8,34].

The second comparative group consisted of 12 patients (8 male, 4 female, age 53–83 years) with surgically resected, poorly differentiated (G3) PDAC diagnosed between 2008–2015. From these 12 cancers, 11 were pT3, and 1 was pT4 stage, respectively. Ten patients with G3 PDAC displayed regional lymph node metastases at surgery. Comparative group PDAC cases were selected randomly among patients with high grade and advanced disease.

Representative tissue blocks of formalin-fixed and paraffin embedded (FFPE) tumor tissue were selected for miRNA extraction from the archives of the departments of pathology in all three involved institutions.

### 2.2. MicroRNA Isolation and Reverse Transcription

For miRNA extraction, one to three 6 µm thick unstained paraffin-embedded tissue sections were obtained and processed using the miRNeasy FFPE kit from Qiagen (Venlo, The Netherlands). Following extraction, two microliters of the isolated RNA were utilized for assessing RNA quantity and purity. This analysis involved measuring the optical density at 260 and 280 nm using the multi-detection microplate reader Synergy HT from BioTek (Winooski, VT, USA), equipped with the Take3 micro-volume plate. RNA integrity was evaluated through denaturing agarose gel electrophoresis, and GeneTools 3.08 software from SynGene (Bengaluru, India) was employed for analysis. Subsequently, reverse transcription was conducted using RevertAid Reverse Transcriptase from Thermo Fisher Scientific (Waltham, MA, USA), following the manufacturer’s protocol. The reaction mixture, containing 1 μg of DNA-free RNA and a mixture of eight stem–loop primers (20 pmol of each stem–loop primer), was utilized for miRNA reverse transcription. These primers were designed using MiRNA Primer Designer software (version 1.0), generously provided by Dr. Fuliang Xie from East Carolina University, USA. The sequences of the stem–loop primers for the pancreatic miRNAs under investigation and the internal control are detailed in Table 1. Additionally, artificial spike RNA (miR-39 from *C. elegans*, 5 × 10^8^ copies) was included in the reaction as an internal control.

### 2.3. Real-Time qPCR

cDNA samples were amplified in duplicate, using the Applied Biosystems 7500 Fast real-time PCR system and Hot FirePol EvaGreen qPCR Mix Plus (Solis BioDyne, Tartu, Estonia), as described in our previous work [17], primers listed in Table 2. The ΔΔCt method was applied to measure the miRNA expression values of interest [35].

### 2.4. Statistical Analysis

All statistical analyses were conducted using S.A.S. release 9.4 (SAS Inc., Cary, NC, USA) and SPSS 25 (IBM Corporation, Armonk, NY, USA), unless otherwise specified. The expression levels of miRNAs in neoplastic and normal cells were compared using a Mann–Whitney test. All hypotheses were tested as two-sided. A significance level of alpha = 0.05 was chosen; thus, *p*-values less than 0.05 were deemed statistically significant.

## 3. Results

*Abnormal miRNA Expression in Pancreatic Cancers*

We investigated the expression of seven miRNAs isolated from FFPE samples of pancreatic adenocarcinomas from 12 patients, as well as 10 samples of UCOGC. The following microRNAs were selected: miR-21, which promotes cell proliferation and may accelerate tumorigenesis [14,36,37]; miR-155, which interacts with tumor protein p53 inducible nuclear protein 1 (TP53INP1) and transforming growth factor β (TGF-β) [31,38,39]; miR-96 and miR-217, which may act as a tumor suppressors, inhibiting the KRAS-signaling pathway [40,41]; as well as miR-148a, miR-196a, and miR-210, which are frequently included in experimental panels for pancreatic carcinoma diagnosis [23,28,42,43,44].

In comparison with normal pancreatic tissue, miR-21 demonstrated increased expression levels, with great variations, from 7-fold up to 44.79-fold in UCOGC samples, and from 3.76-fold up to 53.51-fold in PDAC (Table 3, Table 4 and Table 5; *p* < 0.05). The miR-155 was also overexpressed in UCOGC (from 1.42-fold to 16.63-fold; *p* < 0.05). This miRNA varied more significantly in PDACs, from 0.86-fold to 232.36-fold (Table 3, Table 4 and Table 5; *p* < 0.05). Expression of miR-210 was also significantly upregulated in both types of tumors: up to 90.7-fold in UCOGC, as well as 65.34-fold in PDAC (Table 3, Table 4 and Table 5; *p* < 0.05). However, we did not detect significant differences between tumor and normal tissue for the expression of miR-96 and miR-196a in G3 PDACs (Table 5, *p* > 0.05) and miR-96 in UCOGC, while miR-196a was slightly but significantly downregulated in the latter (*p* < 0.05, Table 5). The expression of miR-148a in the tumor samples was downregulated in both UCOGC and PDAC (Table 3, Table 4 and Table 5; *p* < 0.05). Specifically, miR-217 displayed a substantial decrease, up to one hundred times in both types of tumors (*p* < 0.05, Table 3, Table 4 and Table 5).

**Figure 1:**
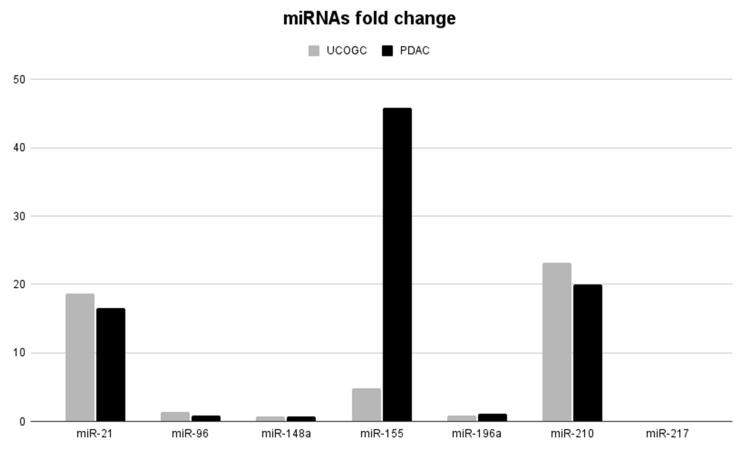
Comparison of miRNA expression fold change in UCOGC and G3 PDAC, shown graphically.

We have not observed significant differences in the expression of the majority of miRNAs between UCOGC and G3 PDAC (*p* > 0.05. Table 5), with the exception of miR-155. UCOGC demonstrated lower mean levels of miR-155 in comparison with PDACs (*p* < 0.05. Table 5). On the other hand, miR-196a was slightly, but significantly, downregulated in UCOGCs (*p* < 0.05. Table 5), whereas significant differences between PDACs and normal tissue samples were not detected (*p* > 0.05. Table 5, Figure 1).

## 4. Discussion

UCOGC is a well-defined WHO subtype of pancreatic cancer that may accompany conventional PDAC or mucinous pancreatic tumors, such as IPMN or mucinous cystic neoplasm (MCN) [2]. UCOGC has been thought to have a poor prognosis [45,46,47], although better than that of conventional PDAC and undifferentiated carcinoma without giant cells [2]. However, there have been descriptions of individual UCOGC cases with favorable clinical courses, including a small subset of “unexpected long survivors” [2,48,49]. To date, programmed death ligand 1 (PD-L1), frequently expressed in UCOGC and rarely in PDAC [50], is the only robust prognostic marker in UCOGC, whereas the PD-L1-negative UCOGC is characterized by long survival and favorable outcome [5,34]. There are two successful clinical trials using anti-PD-L1 treatment in UCOGC described in the literature [51,52].

Of note, there is evidence pointing towards a worse prognosis of UCOGC associated with PDAC compared to pure UCOGC, which has been explained by a higher degree of the epithelial–mesenchymal transitional phenotype [53].

However, little is known about the genomic background and biological rationale for the peculiar morphology and immunohistochemical phenotype of UCOGC. A recent large genomic landscape study analyzing 2568 PDAC cases revealed the canonical mutations of *KRAS* (85.1%), *TP53* (69.1%), *CDKN2A* (35.4%), and *SMAD4* (19.4%) to be the most common. Additionally, mutations in *CDKN2B*, *ARID1A*, *STK11*, *MYC*, *KDM6A,* and *DNMT3A* were among top ten DNA abnormalities [54]. A review by Bazzichetto presented a similar distribution of driver mutations in UCOGC for *KRAS* (70–100%), *TP53* (50–100%), *SMAD4* (10–50%), and *CDKN2A* (25%) as non-canonical possibly oncogenic mutations. *SERPINA3*, *MAGEB4*, *GLI3*, *MEGF8*, *TTN*, and *BRCA2* have been described in a small number of UCOGCs [6]. Our group recently reported 10 *KRAS*, 9 *TP53*, 4 *CDKN2A*, and 1 *SMAD4* mutations when analyzing 13 UCOGC cases [9]. Bergmann et al. identified druggable genomic targets (L1CAM, cyclooxygenase 2, epidermal growth factor receptor) in a high proportion of undifferentiated pancreatic carcinomas, including UCOGC [55]. To the best of our knowledge, except for a single case analysis [56], there are no other studies comparing the spectrum of RNA expression in UCOGC compared to PDAC.

Many cancer pathways in PDACs are regulated by microRNAs. However, little is known about miRNA expression profiles in UCOGC. Aberrant expression levels of miRNAs have been extensively documented in various malignant tumors, playing a crucial role in the formation, development, and prognosis of pancreatic cancers. These miRNAs can generally be categorized into two major groups: oncomirs and tumor suppressors. In pancreatic cancer, a significant number of oncomirs are found to be overexpressed, contributing to disease progression and aggressiveness. Nakata et al. and Eun et al. reported that miR-21, miR-155, and miR-210 were aberrantly expressed in PDAC [22,57]. These miRNAs stimulate PDAC cell proliferation, invasion, and chemoresistance and prevent apoptosis, as well as promote tumor angiogenesis and survival in hypoxic conditions [36,39,58,59,60,61,62] (Table 6).

MiR-21 is among the earliest identified oncomirs and is known for promoting cancer development. It targets nearly 30 genes, including tumor suppressors such as CDK2AP1, Pdcd4, and BCL2 [63]. MiR-21 stimulates pancreatic ductal adenocarcinoma (PDAC) cell proliferation, invasion, and chemoresistance, while also preventing apoptosis [14,15,36,58,68]. MiR-21 has been discovered to activate the epidermal growth factor (EGF) pathway by binding to sprouty RTK signaling antagonist 2 (Spry2). This interaction leads to heightened cell proliferation and triggers downstream signaling pathways such as MAPK/ERK and PI3K/Akt [69,70]. Nakamura et al. investigated exosomal miRNAs in pancreatic juice, finding that miR-21 and miR-155 can be used to differentiate PDAC patients from CP patients [65]. Wang et al. established that elevated serum levels of miR-21 might serve as a predictor for chemoresistance in PDAC [71]. Karasek et al. described the association of poor patient overall survival (OS) and elevated plasma levels of miR-21 [72]. On the other hand, Khan et al. observed worse progression-free survival (PFS) in patients with inoperable PDAC and elevated plasma miR-21, but no association between circulating miR-21 levels and OS was detected [73]. The levels of miR-21 expression show an increase in pancreatic ductal adenocarcinoma (PDAC) and exhibit considerable variation across different research studies [28,29,36,42]. Bloomston et al. observed a median 2.2-fold rise in miR-21 expression levels in formalin-fixed paraffin-embedded (FFPE) tumor samples [28]. In contrast, Zhang et al. identified significantly elevated levels of miR-21 expression, showing an increase of up to 6888-fold in various tumor samples [23]. In this study, the expression level of miR-21 was significantly increased in PDACs, as well as in UCOGCs in comparison with that in normal tissue (*p* < 0.05, Table 5).

MiR-155 acts as an inhibitor of tumor protein 53-induced nuclear protein 1 (TP53INP1) and FOXO3a expression, consequently promoting cell proliferation and malignant transformation [39,59]. Moreover, miR-155 is linked to the JAK/STAT pathway, exerting a negative regulatory effect on SOCS1. This correlation contributes to the enhanced migration and invasion capabilities of PDAC cells [64]. The expression levels of six miRNAs, among them miR-155, in tumor biopsies collected from human pancreatic ductal adenocarcinoma (PDAC) patients were discovered to be significantly associated with a higher risk of lymph node metastasis. This finding not only enhances the accuracy of diagnosing lymph node metastasis, but also, when combined with serum CA19-9 levels [74], MiR-155 has also been associated with advanced tumor stage and poor survival outcomes [26]. Mikamori et al. demonstrated that both OS and disease-free survival (DFS) were significantly shorter in the group with high miR-155 expression in microdissected formalin-fixed paraffin-embedded (FFPE) pancreatic cancer samples [75]. Similarly to miR-21, the expression of miR-155, miR-196a, and miR-10b has been correlated with increased invasiveness and poor OS in pancreatic cancer patients [76]. Pang et al. illustrated that PDAC cells have the ability to induce the differentiation of cancer-associated fibroblasts (CAFs) or CAF-like cells from normal fibroblasts by releasing exosomes containing miR-155. These miR-155-containing exosomes can directly interact with TP53INP1, facilitating this differentiation process [69,77]. Recently published pooled results on the role of increased levels of miR-155 in cancer diagnosis and prognosis showed a remarkable diagnostic value of miR-155 in regards to several cancers, including PDAC [78]. Kim et al. recommended several serum miRNAs, including miR-21 and miR-155, for accurate PDAC diagnosis [79]. In various studies, the overexpression of miR-155 in paraffin-embedded pancreatic ductal adenocarcinoma samples and pancreatic cancer cell lines has been observed, with the fold change ranging from 1.8 to 2.9 [23,28,80]. On the other hand, Zhang et al. reported an up to 52-fold increase in individual cases [23]. In our group of patients, we detected a mean 4.91-fold and 45.8-fold increase in miR-155 expression in UCOGCs and G3 PDACs, respectively (*p* < 0.05, Table 5).

MiR-210 is necessary for tumor angiogenesis, cell cycle regulation, and cancer survival in hypoxic conditions [60,61,62,67]. Research has revealed that miR-210 imposes a negative regulatory influence on EFNA3 expression and actively engages in the PI3K/AKT/VEGFA or Wnt/β-catenin/RHOA pathways. This participation facilitates tumor angiogenesis and enhances cellular permeability. In vivo studies have corroborated that exosomal miR-210 plays a significant role in driving the progression of pancreatic ductal adenocarcinoma [81,82]. Yu et al. analyzed plasma levels of miR-196a and miR-210 with RT-qPCR in a cohort of 31 PDAC patients. High miR-196a expression was associated with poor OS, whereas high miR-210 expression was significantly associated with improved survival [83]. An increase in miR-210 levels has been consistently reported across numerous studies [31,43]. In their study, Greither et al. identified a significant increase of up to 39.9-fold in miR-210 levels in snap-frozen surgical resection specimens [31]. Wang et al. reported a substantial elevation ranging from 2 to 28-fold in miR-210 plasma levels among patients diagnosed with pancreatic PDAC [30]. In this study, the expression level of miR-210 was significantly increased in G3 PDACs, as well as in UCOGCs, in comparison with the level in normal tissue (*p* < 0.05, Table 5).

The miR-196a is an oncomir reported to be frequently dysregulated in PDAC [28,31]. Serum levels of miR-196a could potentially serve as a valuable marker for distinguishing between resectable and unresectable PDAC [84]. Nuclear factor-kappa-B-inhibitor alpha (NFKBIA) is a direct target for miR-196a. This miRNA promotes cancer cell proliferation, migration, and invasion. Bloomston et al. have associated high expression levels of miR-196a in formalin-fixed paraffin-embedded (FFPE) samples obtained from PDAC patients, as measured by microarrays, with shorter OS [28]. Kong et al. similarly reported a correlation between elevated levels of miR-196a measured in the blood sera of pancreatic ductal adenocarcinoma patients and poorer survival outcomes, as well as advanced disease stage [26]. Abnormalities in miR-196a expression have been documented, not only in pancreatic cancer, but also in various other malignancies. [23,30,85,86]. Zhang et al. observed variations in tumor miR-196 expression ranging from 0.35 to 1557-fold [23]. Meanwhile, Wang et al. demonstrated a significant 16.05-fold increase in miR-196 expression levels in plasma samples [30]. We did not find significant differences in miR-196a expression between G3 PDACs and normal tissues (*p* > 0.05, Table 5). On the other hand, this miRNA was slightly but significantly downregulated in UCOGCs samples (*p* < 0.05, Table 5).

The microRNAs miR-96, miR-148a, and miR-217 are considered to be tumor suppressors, and they are inhibited in PDACs. These miRNAs may inhibit cell proliferation and prevent cancer cells chemoresistance, migration, and invasions; additionally, they induce cellular senescence and apoptosis [32,33,40,41], Table 7.

MiR-96 may act as a tumor suppressor, inhibiting the KRAS-signaling pathway [40]. Indeed, the expression of miR-96 in PDAC appears to be inconsistent across various studies, with some reporting downregulation [29,40,43], while others suggest upregulation [28]. On the other hand, we did not find any significant differences in miR-96 expression levels between UCOGCs, G3 PDACs, and normal tissues (*p* > 0.05, Table 5).

MiR-148a targets may affect the cell cycle and apoptosis [32]. The expression of microRNA miR-148a is reported to be downregulated in pancreatic ductal adenocarcinoma as a result of promoter hypermethylation. This phenomenon represents an early event in the process of pancreatic carcinogenesis [91]. Bloomston et al. and Jamieson et al. both measured a significant average decrease in miR-148a expression, with Bloomston et al. observing a 5.5-fold decrease, and Jamieson et al. reporting a 7.14-fold decrease [25,28]. a decrease in miR-148a levels was identified in UCOGC, as well as in G3 PDAC tissue samples (*p* < 0.05, Table 5).

MiR-217 has been shown to inhibit in vitro tumor cell growth, and it acts as a potential tumor suppressor by influencing the Akt/KRAS signaling pathway. Consequently, miR-217 is often found to be downregulated in PDAC [41]. Conversely, miR-217 is typically expressed in normal pancreatic tissue [92], and downregulated in pancreatic dysplasia [93]. In the study by Szafranszka et al., microRNA miR-217 was found to be downregulated by 10-fold [43]. However, Greither et al. determined only a mean 2-fold decrease [31]. Hong et al. discovered that the expression of miR-217 was downregulated by up to 62.5-fold in PDACs [29]. Both UCOGC and G3 PDAC samples demonstrated a significant downregulation of miR-217, with an up to-100-fold decrease (*p* < 0.05, Table 5).

In the current study, no significant differences in the expression of the majority of miRNAs were observed between UCOGC and G3 PDAC (*p* > 0.05, Table 5), with the exception of miR-155. UCOGC demonstrated distinctly lower mean levels of miR-155 in comparison with those of the PDACs (*p* < 0.05, Table 5). MiR-155 is an oncomir overexpressed in early pancreatic adenocarcinoma precursors and invasive PDAC [38]. High expression of this miRNA was associated with lymph node metastasis in PDAC patients [94,95]. The microRNA miR-155 was found to induce epithelial–mesenchymal transitions (EMT) in breast, liver, and kidney cancer [96,97,98,99]. MiR-155 promotes (EMT), as well as regulates cancer cell invasion and migration, by modulating the STAT3 signaling pathway in pancreatic cancer cells [64]. Mattiolo et al. reported that EMT is more frequently activated in undifferentiated carcinomas (UC) (10/10 cases) than in UCOGC (8/16 cases). Furthermore, in UCOGC, EMT was activated with a higher frequency in cases with an associated PDAC component. Snai2 was the most frequently and strongly expressed marker of EMT in both tumor types (10/10 UC, 8/16 UCOGC), and its expression was higher in UC than in UCOGC [53]. Therefore, our results indicating that poorly differentiated G3 PDACs demonstrated higher levels of miR-155 in comparison with UCOGC are in concordance with the findings mentioned above. On the other hand, our work is of a preliminary nature. We believe that a cohort of 10 UCOGC patients is not enough for conducting statistical analysis on the correlation between miRNA fold change and patient outcomes. Consequently, future investigations involving a larger number of patients are warranted. Additionally, our study only examined seven miRNAs. Therefore, analyzing the entire miRNome, using RNA microarray or sequencing techniques, may be instrumental in identifying miRNAs that are highly beneficial for PDAC and UCOGC diagnostics, therapy, or predicting patient outcomes.

## 5. Conclusions

We have demonstrated that UCOGC, as well as PDAC, display similar pattern of changes in miRNAs expression in comparison with those of normal tissues. Oncomirs, including miR-21, miR-155, and miR-210, were significantly upregulated. Tumor suppressors, such as miR-148a and miR-217, were downregulated in both UCOGC and PDAC. We did not find any significant differences between cancerous and normal tissues for the expression levels of miR-96 and miR-196a in PDAC, whereas miR-196a was downregulated in UCOGC. We have not observed significant differences in the expression of the majority of miRNAs between UCOGC and PDAC, with the exception of miR-155.

## Figures and Tables

**Table 1:** Stem–loop primers for the miRNAs.

miRNA Name:	Stem–Loop Primer Sequence:
miR-39 *C. elegans*	GTCGTATCCAGTGCAGGGTCCGAGGTATTCGCACT- GGATACGACTATTAC
mir-21	GTCGTATCCAGTGCAGGGTCCGAGGTATTCGCAC- TGGATACGACTCAACA
miR-96	GTCGTATCCAGTGCAGGGTCCGAGGTATTCGCACTGGATA- CGACAGCAAAAATGTG
miR-148a	GTCGTATCCAGTGCAGGGTCCGAGGTATTCGCACTG- GATACGACAGTCGGAG
miR-155	GTCGTATCCAGTGCAGGGTCCGAGGTATTCGCACTGGATA- CGACACCCCTATCACG
miR-196a	GTCGTATCCAGTGCAGGGTCCGAGGTATTCGCACTGGATA- CGACCCCAACAACATG
miR-210	GTCGTATCCAGTGCAGGGTCCGAGGTATTCGCACTGGATAC- GACTCAGCCGCTGTC
miR-217	GTCGTATCCAGTGCAGGGTCCGAGGTATTCGCACTGGATA- CGACTCCAATCAGTTC

**Table 2:** Real-time qPCR primers.

Primer Name:	Primer Sequence:
Universal primer	ATCCAGTGCAGGGTCCGAGG
mir-39 *C. elegans*	GCGGCGGAGCTGATTTCGTCTTG
mir-21	GCGGCGGTAGCTTATCAGACTG
miR-96	GCGGCGGTTTGGCACTAGCAC
miR-148a	GCGGCGGAAAGTTCTGAGACACTCC
miR-155	GCGGCGGTTAATGCTAATCGTG
miR-196a	GCGGCGGTAGGTAGTTTCATGTTG
miR-210	GCGGCGGCTGTGCGTGTGACAG

**Table 3:** MicroRNAs expression fold change and clinical data in UCOGC.

ID	Clinical Data						miRNA						
	Diagnosis	Sex	Age	Stage	Survival (Months)	Status	miR-21	miR-96	miR-148a	miR-155	miR-196a	miR-210	miR-217
1	UCOGC	M	73	pTXN1	4	Dead	13.25	0.42	0.48	6.34	1.48	45.88	0.06
2	UCOGC	F	76	pT3N0	0.1	Dead	12.42	0.26	0.61	3.73	0.96	14.84	0.03
3	UCOGC	M	61	pT3N0	9	Dead	10.92	0.56	0.39	3.35	0.91	4.49	0.026
4	UCOGC	M	53	pT3N1	58	Alive	16.68	0.27	1.06	2.18	0.43	7.92	0.02
5	UCOGC	M	63	pT1cN0	57	Alive	44.79	0.38	0.76	5.61	0.89	39.98	0.08
6	UCOGC	M	67	pT3N1	1	Dead	7	1.99	0.79	1.42	0.96	4.06	0.17
7	UCOGC + PDAC	M	63	pT2N1	6	Dead	15.79	0.92	0.17	16.63	0.44	90.7	0.07
8	UCOGC + IPMN	F	50	pT1bN0	86	Alive	34.39	2.21	0.46	3.19	0.45	15.88	0.06
9	UCOGC	M	59	pT2N0	168	Dead	11.62	6.22	2.12	1.69	1.14	4.39	0.05
10	UCOGC + PDAC	M	75	pT3N1	6	Dead	19.39	0.14	0.37	4.92	0.75	3.99	0.01

Min and max values are shown in red.

**Table 4:** MicroRNAs expression fold change and clinical data in G3 PDAC.

ID	Clinical Data						miRNA						
	Diagnosis	Sex	Age	Stage	Survival (Months)	Status	miR-21	miR-96	miR-148a	miR-155	miR-196a	miR-210	miR-217
1	PDAC	M	69	pT3N1	23	Dead	7.83	0.456	0.68	3.46	1.372	11.17	0.025
2	PDAC	M	68	pT3N1	18	Dead	13.16	0.824	1.4	16.62	0.797	28.07	0.105
3	PDAC	M	83	pT3N1	29	Dead	3.76	0.396	0.847	2.27	0.55	3.9	0.083
4	PDAC	F	71	pT3N1	7	Dead	11.11	0.663	1.27	10.58	1.069	7.96	0.031
5	PDAC	M	63	pT3N1	45	Dead	53.51	1.45	0.137	80.67	1.51	26.88	0.001
6	PDAC	M	59	pT3N1	15	Dead	8.08	0.55	0.178	0.86	1.04	3.61	0.006
7	PDAC	M	60	pT3N1	9	Dead	4.61	1.91	2.16	7.74	1.205	9.44	0.075
8	PDAC	F	66	pT3N1	23	Dead	12.28	1	0.493	9.18	1.28	13.81	0.125
9	PDAC	M	64	pT3N1	12	Dead	5.9	0.41	0.116	54.26	0.996	41.98	0.002
10	PDAC	F	53	pT4N1	11	Dead	14.96	0.76	0.232	232.36	1.09	5.9	0.031
11	PDAC	M	67	pT3N0	27	Dead	28.43	1.497	0.519	121.05	1.42	21.35	0.236
12	PDAC	F	77	pT3N0	9	Dead	35.4	0.792	0.053	10.56	1.4	65.34	0.103

Min and max values are shown in red.

**Table 5:** Comparison of miRNAs expression fold change in UCOGC and G3 PDAC. Data are presented as means ± standard deviation (SD). Statistically significant differences between cancer and normal tissue samples are shown in bold. Significant differences between studied types of pancreatic cancers are highlighted in red. The Mann–Whitney test was used for the calculations. *p*-values of *p* < 0.05 were considered statistically significant.

Cancer Type	miRNAs Fold Change						
	miR-21	miR-96	miR-148a	miR-155	miR-196a	miR-210	miR-217
UCOGC	**18.62** **± 8.539**	1.34 ± 1.281	**0.72** **± 0.369**	**4.91** **± 2.775**	**0.84** **± 0.259**	**23.21** **±** **21.384**	**0.05** **± 0.031**
PDAC	**16.58** **± 11.263**	0.89 ± 0.381	**0.67** **± 0.498**	**45.8** **± 50.856**	1.14 ± 0.22	**19.95** **± 13.977**	**0.06** **± 0.052**

**Table 6:** Target genes for the oncomirs in pancreatic tumors.

Oncomirs (Upregulated)	miRNA Targets	miRNA Enhances	References
miR-21	CDK2AP1, Pdcd, BCL2, PTEN, and almost 30 genes	proliferation, invasion, chemoresistance, tumor survival	[14,15,63,64]
miR-155	*TP53INP1*, *FOXO3a*, and *SOCS1*	proliferation, transformation, migration, invasion	[38,59,65]
miR-196a	*NFKBIA*	proliferation, migration, invasion	[66]
miR-210	*Ephrin-A3*, *MNT*	proliferation, angiogenesis, tumor growth, survival	[26,67]

**Table 7:** Tumor-suppressing miRNAs in pancreatic cancer.

Tumor Suppressors (Downregulated)	miRNA Targets	miRNA Inhibits	References
miR-96	*KRAS*	proliferation, migration	[87,88,89]
miR-148a	*PHLDA2*, *LPCAT2,* and *AP1S3*	proliferation, migration, invasion	[32,90]
miR-217	*KRAS*, *SIRT1*	proliferation, migration	[37,65]

## Data Availability

All data supporting reported results can be found in the authors’ institutions or in public repositories.
